# Editorial: Graphene-Enhanced Electrochemical Sensing Platforms

**DOI:** 10.3389/fchem.2021.815981

**Published:** 2021-12-02

**Authors:** Li Fu, Fatemeh Karimi, Aimin Yu

**Affiliations:** ^1^ Key Laboratory of Novel Materials for Sensor of Zhejiang Province, College of Materials and Environmental Engineering, Hangzhou Dianzi University, Hangzhou, China; ^2^ Department of Chemical Engineering and Energy, Quchan University of Technology, Quchan, Iran; ^3^ Department of Chemistry and Biotechnology, Faculty of Science, Engineering and Technology, Swinburne University of Technology, Hawthorn, VIC, Australia

**Keywords:** sensor, graphene, cancer, soil, antioxidant, drug detection, drug resisitance

Graphene is a two-dimensional carbon material with very wide applications. Its emergence has had a very significant impact on the field of electrochemical sensing. The most direct impact is about the large number of electrochemical sensors modified with graphene materials have been reported. The purpose of this Research Topic is to attract scientists from different fields to provide individual research on graphene electrochemical sensors. This Research Topic attracted a total of 12 papers, including eight research articles and four mini-reviews. Not surprisingly, this topic has attracted research efforts in different fields, including drug detection, investigation of antioxidant properties, analysis of soil organic matter content and characterization of antimicrobial properties.

The detection of cancer indicators is the most frequent research direction in this Research Topic. Zhang et al. summarized the development of carbon nanomaterials for electrochemical analysis of gastric cancer markers, with particular emphasis on the important work of graphene in recent years. Carcinoembryonic antigen (CEA), carbohydrate antigen (CA) 125, CA19-9, CA72-4 and several miRNAs were presented in detail as the most important gastric cancer markers. Wei et al. summarized the recent development of graphene-based electrochemical sensors for detecting hematological malignancies-associated biomarkers. They highlighted electrochemical sensors in RNA biomarkers and protein-based markers. In addition, this mini-review specifically contains label-free electrochemical sensors for the detection of DNA and CTC markers in blood tumors. A mini-review was also contributed by (Xu et al.). They focused on the analytical application of graphene electrochemical sensors in pancreatic cancer detection. They also discussed the use of CEA detection in pancreatic cancer screening. Due to the specificity of pancreatic cancer, they also discuss the application of graphene-assisted electrochemical sensors in mutant K-Ras gene detection. In addition to the review, this Research Topic also attracted a research paper on cancer indicator detection. Wang et al. reported an integrated electrode system on an FR-4 glass fiber. Graphene electrode has been used as a working electrode. The proposed electrochemical sensor can be used for linear sensing of CEA in the range of 0.2–15.0 ng/ml with a limit of detection of 0.085 ng/ml. From the above studies, it is clear that there is no particularly strong specificity between cancer indicators and cancer, for example, CEA can be used as an indicator for many different cancers. Therefore, for the diagnosis of cancer, a combination of multiple indicators is often required. Therefore, how to establish an electrochemical sensor for multi-indicator detection is an important direction for this technology in the future. This Research Topic not only attracts detection for cancer indicators, but also for cancer drugs. Wu et al. reported a biosynthesized graphene-silver nanocomposite for imatinib detection.

The detection of indicators has a very important place in clinical research. In addition to cancer indicators, troponin I and blood glucose are very important indicators in the clinical diagnosis of myocardial infarction and diabetes, respectively. In this Research Topic, Li et al. reported a silver nanoparticles/MoS_2_/reduced graphene oxide electrochemical sensor for cardiac troponin I detection. Meanwhile, another mini-review summarizes the application of electrochemical POC sensors in blood glucose detection (Li et al.). The POC blood glucose sensor is a product that has been commercialized, but still has some application disadvantages. Graphene may be able to provide new solutions to these problems.

Graphene has become a platform for evaluating microbial resistance due to its excellent electrical properties and its two-dimensional structure that allows microorganisms to be sequestered. This Research Topic has attracted two articles in this field. Li and Sun reported a graphene-assisted electrochemical sensor for antibiotic resistance detection in *Escherichia coli.* Duan et al. also reported an electrochemical sensor for drug resistance of *Escherichia coli.* However, methylene blue was used as a probe in this work.

In addition to the common electrochemical sensors mentioned above, this Research Topic has attracted three interesting works related to the agricultural and phytological fields. Yue et al. used an electrochemical sensor for peroxidase evaluation in herbal medicines. The reduction of hydrogen peroxide by a graphene-assisted sensor was used as a signal. Yan et al. reported a graphene oxide-embedded hydrogel for antioxidant activity evaluation of *Scutellaria baicalensis.* Liu et al. reported a pioneer work. For the first time, they used graphene-modified electrodes for the detection of organic matter content in the soil.

